# Norovirus Genotype Profiles Associated with Foodborne Transmission, 1999�?"2012

**DOI:** 10.3201/eid2104.141073

**Published:** 2015-04

**Authors:** Linda Verhoef, Joanne Hewitt, Leslie Barclay, Sharia Ahmed, Rob Lake, Aron J. Hall, Ben Lopman, Annelies Kroneman, Harry Vennema, Jan VinjA(c), Marion Koopmans

**Affiliations:** National Institute for Public Health and the Environment, Bilthoven, the Netherlands (L. Verhoef, A. Kroneman, H. Vennema, M. Koopmans);; Institute of Environmental Science and Research, Porirua, New Zealand (J. Hewitt, R. Lake);; Centers for Disease Control and Prevention, Atlanta, Georgia, USA (L. Barclay, S. Ahmed, A.J. Hall, B. Lopman, J. VinjA(c));; Erasmus Medical Center, Rotterdam, the Netherlands (M. Koopmans)

**Keywords:** norovirus, genotype, transmission, surveillance, foodborne, epidemiology, virology, gastroenteritis, communicable diseases, outbreaks, attribution, viruses, enteric infections

## Abstract

Worldwide, noroviruses are a leading cause of gastroenteritis. They can be transmitted from person to person directly or indirectly through contaminated food, water, or environments. To estimate the proportion of foodborne infections caused by noroviruses on a global scale, we used norovirus transmission and genotyping information from multiple international outbreak surveillance systems (Noronet, CaliciNet, EpiSurv) and from a systematic review of peer-reviewed literature. The proportion of outbreaks caused by food was determined by genotype and/or genogroup. Analysis resulted in the following final global profiles: foodborne transmission is attributed to 10% (range 9%%�?"11%) of all genotype GII.4 outbreaks, 27% (25%�?"30%) of outbreaks caused by all other single genotypes, and 37% (24%%�?"52%) of outbreaks caused by mixtures of GII.4 and other noroviruses. When these profiles are applied to global outbreak surveillance data, results indicate that �%^14% of all norovirus outbreaks are attributed to food.

## Abstract 2

Foodborne transmission accounts for 10% of outbreaks caused by GII.4, 27% by all other single genotypes, and 37% by mixtures of GII.4 and others

Noroviruses are a leading cause of gastroenteritis worldwide. They belong to the family *Caliciviridae* and consist of an �%^7.5-kb genome in 3 open reading frames (ORFs). The first ORF (ORF1) encodes a polypeptide; ORF2 encodes the viral capsid protein (VP1); and ORF3 encodes a minor structural protein (VP2). Noroviruses are classified into at least 6 genogroups, GI�?"GVI (*1*). According to a recent unified proposal for nomenclature, genogroups are further subdivided into at least 38 genetic clusters (genotypes) (*2*). Noroviruses are environmentally stable (*3*) and can be transmitted by different routes (e.g., foodborne, personborne, waterborne, and environmental). Determining the transmission route during an outbreak investigation is complicated because transmission can occur by multiple routes in a single outbreak. After primary introduction of the virus through food, secondary person-to-person and environmental transmission can rapidly take over, making it hard to trace the disease back to contaminated food. Another complexity is that foodborne transmission can follow different routes as well; food can be contaminated during production (*4*) or during handling by an infected food handler (*5*).

Different exposure attribution methods (i.e., epidemiologic investigations, microbiological typing/subtyping, intervention studies, and expert elicitations) have been used to estimate the foodborne proportion of the overall disease incidence caused by a pathogen. Each approach has its advantages and disadvantages, and therefore the use of multiple methods has been recommended (*6*). Information about pathogen strain or subtypes may be of value for attribution but is dependent on substantial amounts of contextual data. For example, a method commonly used to attribute *Salmonella* spp. infections to a specific source uses strain collections representative of the pathogen in each of these sources (*7*).

For noroviruses, genogroup-specific differences have been reported with regard to environmental persistence (*8*), sensitivity to removal (*9*), and binding to receptors (*10*). These biological differences may underpin strain-specific epidemiologic patterns, suggesting a potentially useful approach for norovirus attribution. Such an approach was recently developed in a norovirus attribution study, which showed that the proportion of foodborne and person-to-person outbreaks differed between genotypes; the GI genotypes were more likely to be foodborne, and the II.4 genotype was more likely to be personborne (*11*). These findings indicate that genotype profiles may help distinguish which outbreaks are more likely to be foodborne than personborne. Also, a recent study on norovirus outbreaks in the United States showed that GI.3, GI.6, GI.7, GII.3, GII.6, and GII.12 were the norovirus genotypes most often associated with foodborne outbreaks and that, of the outbreaks with a known transmission route, 16% were foodborne (*12*). Norovirus infections, however, are a global problem, and efforts are under way to estimate the global social and economic costs of foodborne norovirus illness (*13*,*14*). To estimate the proportion of outbreaks attributed to foodborne transmission from a global perspective, we used aggregated norovirus outbreak data and genotyping information from different outbreak surveillance systems and from peer-reviewed literature. 

## Methods

Data from 4 sources were available for comparison: 3 laboratory-based norovirus outbreak surveillance network databases and 1 systematic review of norovirus outbreaks in the peer-reviewed published literature. Laboratory surveillance networks aim to link norovirus outbreaks that may be caused by common sources (such as food), monitor genotype trends, and identify emerging norovirus strains. Relevant data from these networks included transmission route(s) and norovirus genotyping performed by sequence analysis of the polymerase (ORF-1) and capsid (VP1/ORF-2) regions of the norovirus genome. Polymerase and capsid sequences were not available for all samples, requiring separate analyses.

### Databases

#### Foodborne Viruses in Europe Network/Noronet

The Foodborne Viruses in Europe (FBVE) network was started in 1999 as a European Union�?"funded combined laboratory and epidemiological network of 13 European countries sharing norovirus outbreak data (*15*). Since 1999, the FBVE network has maintained a joint database in which members have shared their data. In 2009, Noronet was started as a continuation of the FBVE network (*13*). Noronet is an informal network of scientists working in public health institutes or universities that share virologic, epidemiologic, and molecular data on norovirus. The network now includes laboratories in countries outside Europe. Although all laboratories use PCR-based methods for norovirus detection, the detected parts of the norovirus genome may differ between laboratories; consequently, genotyping is not necessarily based on the same part of the genome. Sequence data shared in the FBVE/Noronet database include regions A and B (polymerase) and/or regions C and D (capsid) of the genome (*15*). Data from 5,583 outbreaks that occurred from January 1999 through December 2012 are included in the analyses.

#### CaliciNet

The US Centers for Disease Control and Prevention developed CaliciNet in 2009 (*16*). CaliciNet is a national norovirus outbreak surveillance network of public health and food regulatory laboratories that submit epidemiologic information and PCR-based norovirus detection and typing information, including regions C and D (capsid) sequences of the genome, to a national database by using standardized protocols (*12*,*16*). As of November 2013, participants from 32 public health laboratories in 28 states have been certified to participate in CaliciNet. Data from 3,094 outbreaks reported from September 2009 through December 2012 are used in the analyses.

#### Institute of Environmental Science and Research�?"EpiSurv

In New Zealand, gastroenteritis outbreaks are reported by public health units that submit epidemiologic information to the EpiSurv database, a surveillance system operated by the Institute of Environmental Science and Research (ESR) for the New Zealand Ministry of Health. As part of the New Zealand norovirus surveillance, the ESR Norovirus Reference Laboratory conducts norovirus detection testing by using PCR-based methods (*17*) followed by genotyping of at least 1 case per outbreak. Sequence data are obtained from region B and/or region C of the genome (*18*). Combined data from the ESR Norovirus Reference Laboratory and EpiSurv are summarized in annual outbreak reports (https://surv.esr.cri.nz/surveillance/annual outbreak). Data from 819 outbreaks reported from January 2008 through December 2012 are included in the analyses.

#### Systematic Literature Review

In 2012, Matthews et al. (*19*) published results of a systematic review of all norovirus outbreaks reported in the scientific literature from 1993 through 2011. We updated this systematic review to include norovirus outbreaks published in 2012 (Technical Appendix). During September 2012, we searched the literature in the EMBASE, Medline, Web of Science, and Global Health databases for the term �?onorovirus�?? and related terms (*19*). Two independent reviewers screened titles and abstracts for relevance. We included articles published in 2012 that fit the following criteria designated by Matthews et al.: 1) full article, 2) published in English, 3) describes human norovirus outbreaks, 4) used PCR-based diagnostics for at least 1 case per outbreak (*19*). We excluded articles describing sporadic norovirus cases and articles describing outbreaks among immunocompromised patients (e.g., transplant recipients). Separate outbreak-level data were extracted from articles reporting multiple outbreaks, but the articles�?(tm) citations were not referenced for outbreak reports. Genogroup and genotype information (and if available, the PCR target region) was extracted.

### Definitions

#### Transmission Routes

To determine the proportion of outbreaks that were caused by foodborne transmission of the virus (hereafter called the foodborne proportion) we accepted the route(s) assigned in the various databases to each outbreak, based on previous investigation of the FBVE database (*11*) and based on the local public health investigation and with the assistance of standardized guidance materials to provide some consistency (http://www.cdc.gov/mmwr/preview/mmwrhtml/rr6003a1.htm). If the primary or secondary mode of transmission was reported as foodborne, this outbreak was considered a foodborne outbreak (includes foods contaminated during production or during handling). If person-to-person transmission was reported to be one of the possible routes, the outbreak was considered a person-to-person outbreak. We excluded outbreaks for which transmission route was listed as environmental or waterborne only (1 for ESR-EpiSurv, 0 for CaliciNet, 68 for FBVE/Noronet).

#### Genotype Profiles

Each of the surveillance databases used genotypes as previously proposed (*2*). For the purpose of attribution, the proportion of outbreaks listed as being transmitted by the foodborne route was calculated for each polymerase genotype (P-type) or capsid genotype (C-type) individually. The proportions of all individual genotypes that were transmitted by the foodborne route, on the basis of a single data source, were termed genotype profiles and were aggregated into categories of profiles according to the analyses described below.

### Analyses

#### Comparison of Data to Test Robustness of Genotype Profiles

First, to test whether the genotype profile�?"based data from 1999�?"2012 could be generalized, we analyzed genotype data for differences in time (i.e., yearly). This analysis for robustness over time was performed for the FBVE/Noronet database only. Second, to test whether the genotype profiles based on these 2 genomic regions (P-type and C-type) for typing could be combined, we analyzed genotypes based on polymerase sequences if they matched with genotype profiles based on capsid sequences. Because the FBVE and ESR-EpiSurv data had capsid and polymerase sequences available, the comparison was performed for these datasets. Third, we tested whether the analysis by individual genotypes could be aggregated into larger categories on the basis of how well their foodborne proportions corresponded. For this purpose, the profile of each individual genotype was compared with a profile consisting of sequences that belonged to 1 of 4 categories (genogroup aggregation): GI, GII genotype 4 (GII.4), GII but not GII.4 (GIInon4), and mixed (genotypes belonging to multiple genogroups). Categories were further aggregated into larger categories if the aggregation did not significantly affect the results. Thus, aggregation into categories was performed in the following 3 steps: 1) if the foodborne proportions of an individual genotype did not statistically differ from 1 of the 4 categories, genotypes were merged into this category; 2) if categories did not statistically differ from each other, these categories were merged into larger categories; and 3) if profiles of different data sources did not statistically differ (on the basis of their CIs), these profiles were merged and considered as a profile from 1 data source.

#### Comparison of Worldwide Data and Estimating the Foodborne Proportion 

We compared the outcomes after the genotypes were aggregated into the proposed categories of all 4 datasets. We used the resulting profiles to estimate the foodborne proportion among outbreaks for which genotype was known but transmission mode was not known and thereby to estimate the foodborne proportion of norovirus outbreaks in different parts of the world.

#### Statistical Analyses

For each genotype in the surveillance systems, we estimated the fraction of outbreaks for which origin was known to be foodborne or person-to-person on the basis of the foodborne proportion of all foodborne plus person-to-person outbreaks for each genotype. We used the estimated proportion of foodborne outbreaks of all foodborne plus person-to-person outbreaks in each genotype to estimate the probability that outbreaks with unknown transmission mode were foodborne. We calculated 95% CIs by using the Monte Carlo simulation with 10,000 random draws from the I� distributions, which are the posterior probabilities of the proportions (*20*). If 95% CIs overlapped, the genotypes were considered not statistically significantly different and genotypes were aggregated into profiles of larger groups. Data were analyzed by using SAS version 9.3 (SAS Institute, Inc., Cary, NC, USA).

## Results

**Figure 1:**
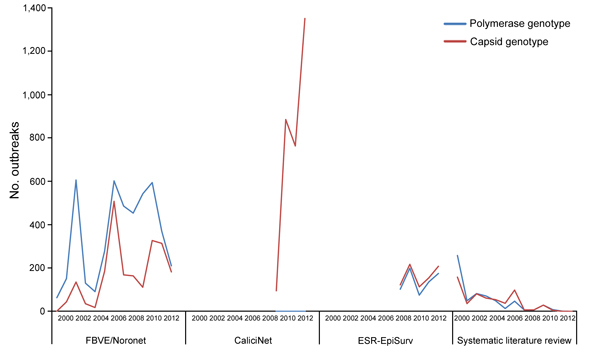
Norovirus data for which the genotyped region and transmission mode were reported in different surveillance systems (FBVE/Noronet, 5,583 outbreaks in 22 countries (1999�?"2012); CaliciNet, 3,094 outbreaks in 1 country (2009�?"2012); ESR-EpiSurv, 818 outbreaks in 1 country (2008�?"2012); and systematic literature review, 808 outbreaks in 61 countries (1993�?"2011). ESR, Institute of Environmental Science and Research; FBVE, Foodborne Viruses in Europe.

**Figure 2:**
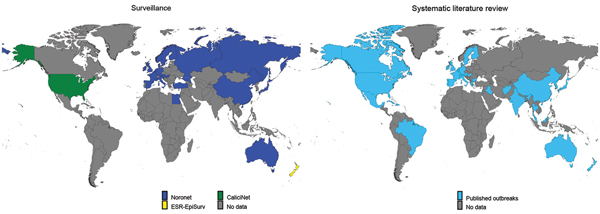
Countries from which norovirus outbreak reports were included in analyses of norovirus genotype profiles associated with foodborne transmission, according to Foodborne Viruses in Europe/ Noronet (1999�?"2012), CaliciNet (2009�?"2012), ESR-EpiSurv (2008�?"2012), or systematic literature review (1993�?"2011). ESR, Institute of Environmental Science and Research.

The FBVE/Noronet database included 5,583 norovirus outbreaks reported from 22 countries during 1999�?"2012 (Figures 1, 2). Of these, C-type sequence information was available for 4,580 outbreaks, P-type for 2,195 outbreaks, and both types for 1,192 outbreaks. The CaliciNet database included information about 3,094 outbreaks that occurred in the United States during 2009�?"2012; C-type sequence information was available for all outbreaks. The ESR-EpiSurv database included 818 outbreaks reported from New Zealand during 2008�?"2012, of which C-type and P-type information was available for 813 and 685, respectively. Our updated systematic review (Technical Appendix) provided information on 966 norovirus outbreaks, of which genotype and transmission mode information was available for 608 (127 C-type, 107 P-type, 374 both) from 61 countries during 1983�?"2010 (Figures 1, 2). Our updated systematic literature search yielded reports of 320 outbreaks in Japan, 113 in the United States, 500 in other countries, and 18 in multiple countries; country information was missing for 15 outbreaks.

### Robustness of Genotype Profiles

Initial analysis showed substantial differences for the estimated proportion of foodborne outbreaks caused by each genotype, depending on the dataset used, the time of reporting, and the method of typing, most likely because of low numbers of outbreaks for some genotypes (Technical Appendix Table). These differences were no longer observed when the data were aggregated into 4 categories (GI, GII.4, GIInon4, and mixed genotype outbreaks) and later into 3 categories (GII.4, all other single genotypes, and mixed genotype outbreaks).

**Figure 3:**
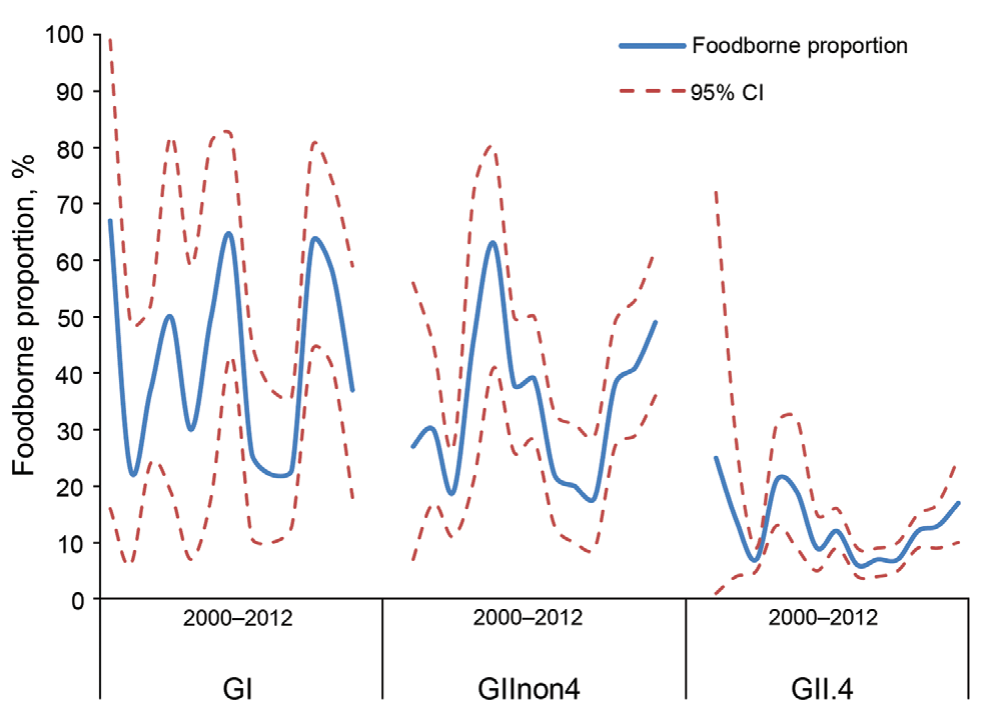
Genotype profiles. Foodborne proportion per genotype group per year, as reported to Foodborne Viruses in Europe/Noronet, with polymerase genotypes (n = 4,580) or, if missing, capsid genotypes (n = 1,003).

With respect to time trend, the comparison of the yearly profiles based on FBVE/Noronet data showed variation in the proportions of outbreaks with foodborne origin per category over time (Figure 3). Because of this finding, together with the knowledge that norovirus is known for its emerging new variants (*13*), the synchronization of time frames of different surveillance databases was considered necessary.

Our analyses showed differences in foodborne proportions for outbreaks for which the C-type was determined and for outbreaks for which the P-type was determined (Technical Appendix Table). This difference is possibly a surveillance artifact caused by multiple countries contributing to FBVE/Noronet, because this effect was not seen in the ESR-EpiSurv profiles (Technical Appendix Table), in which P and C types were both available for 685 outbreaks. For this reason the profiles for each genomic region were kept separately.

With respect to the genotypes versus aggregated categories, for FBVE/Noronet and ESR-EpiSurv data, aggregation of individual genotypes into the GI and GIInon4 categories showed that these cannot be statistically distinguished but that they differ from the categories of the GII.4 genotype and mixed outbreaks. GII.4 and mixed outbreaks show statistical difference and need to be treated as separate categories (Technical Appendix Table). For CaliciNet and the updated systematic review data, results were similar to those of FBVE/Noronet and ESR-EpiSurv but with one difference (i.e., that mixed outbreaks could not be statistically distinguished as a separate group in the CaliciNet dataset). On the basis of these results, we used the following criteria for subsequent profile comparisons: 1) assignment into 3 categories (GII.4, all other single genotypes, and mixed genotypes); 2) separate analysis of data based on P-type and C-type; and 3) synchronized periods for different surveillance systems (2009�?"2012).

### Database Comparisons 

For outbreaks reported during 2009�?"2012, the P-type profiles from the New Zealand surveillance system, the European surveillance system, and from other countries contributing to Noronet were comparable (Table). Attribution profiles from CaliciNet showed similar values for the categories of GII.4 and nonGII.4 outbreaks but different values for the group of mixed outbreaks. However, this finding was based on 31 (1.0%) of 3,094 outbreaks. Because of this low number and the knowledge that how these outbreaks are ascertained in the respective surveillance systems might differ, we considered aggregation of the mixed outbreaks category of the 3 surveillance databases justified. This consideration is strengthened by the finding that foodborne proportions are similar for the other 2, far more common, categories (GII.4, nonGII.4).

**Table:** Proportion of foodborne outbreaks per category, estimated according to different databases for 2009�?"2012*

Genotype	Database				
	FBVE/Noronet, n = 1,715	ESR-EpiSurv, �?"n = 584	CaliciNet, �?"n = 3,094	Systematic literature review, n = 8	Global profile, �?"n = 5,393
GII.4	*0.07 (0.06�?"0.09)*	*0.09 (0.06�?"0.11)*	*0.12 (0.10�?"0.14)*	*0.12 (0.00�?"0.40)*	0.10 (0.09�?"0.11)
Other genotypes	*0.31 (0.25�?"0.37)*	*0.25 (0.17�?"0.33)*	*0.26 (0.23�?"0.30)*	*0.67 (0.16�?"0.97)*	0.27 (0.25�?"0.30)
Mixed outbreaks	*0.75 (0.48�?"0.94)*	*0.50 (0.20�?"0.75)*	**0.16 (0.05�?"0.33)**	CNBC	0.37 (0.24�?"0.52)
Foodborne proportion�?	0.12	0.13	0.16	CNBC	0.14

*FBVE/Noronet polymerase profile was used to compare with ESR-EpiSurv polymerase, systematic literature review polymerase, and CaliciNet capsid profiles. CNBC, could not be calculated; ESR, Institute of Environmental Science and Research; FBVE, Foodborne Viruses in Europe. Estimates in italics do not statistically differ from the FBVE/Noronet polymerase profile as a reference; estimates in boldface do statistically differ. �?"�? Proportion of outbreaks with an unknown mode of transmission estimated to be attributed to food.

The profiles resulting from the updated systematic review differed, but not significantly, probably because only 8 outbreaks were selected for the included time span of 2009�?"2012. This finding resulted in no distinguishable categories, and therefore this dataset was excluded from further calculations. On the basis of this analysis, for food attribution purposes, the profiles from the United States, New Zealand, Europe, and other countries contributing to Noronet can be merged into a global profile (Table).

### Foodborne Attribution of Norovirus Outbreaks

Applying the profiles per surveillance database, the proportion of outbreaks attributed to foodborne transmission varied slightly (12% for FBVE/Noronet, 13% for ESR-EpiSurv, and 16% for CaliciNet), the aggregated global estimate was 13.7%. Overall, 10% (range 9%�?"11%) of all GII.4 norovirus outbreaks, 27% (25%�?"30%) of outbreaks caused by all other single genotypes, and 37% (24%�?"52%) of outbreaks with mixtures of GII.4 and other genotypes were attributed to foodborne transmission. Applying the global attribution profile to all outbreaks reported worldwide in surveillance systems in 2009�?"2012, of the outbreaks with an unknown mode of transmission, 193 (14.5%) of 1,332 outbreaks could be attributed to food.

## Discussion

This analysis of aggregated surveillance datasets of norovirus outbreaks suggests that genotyping can provide useful information for attribution. At 14%, the estimated proportion of outbreaks attributed to foodborne transmission is comparable across the 3 independent datasets. We found that the proportion of outbreaks caused by foodborne transmission was lower for outbreaks caused by GII.4 norovirus than for those associated with all other genotypes (Table). This finding is consistent with previous findings (*11*). GII.4 viruses are notorious for their potential to spread easily from person to person and their rapid evolution (*21*). GII.4 viruses are more often described as causing outbreaks in (semi-)closed settings (*12*,*18*,*22*�?"*24*), implying a higher proportion of person-to-person spread as well. Although proportionally low, the absolute contribution of GII.4 foodborne outbreaks to the social and economic costs of outbreaks caused by noroviruses is considerable, given the numbers of outbreaks caused by this genotype.

Although the calculations for mixed infections were based on low numbers, mixed norovirus infections were less frequently associated with foodborne outbreaks in the United States than in the other countries for which data were available. Mixed infections have been associated with sewage-related outbreaks (*25*). Further research is needed to confirm whether our findings reflect true differences or differences in laboratory and investigative practices.

The use of internationally collected surveillance data and published outbreak reports is potentially associated with different kinds of biases. Therefore, our study has some limitations. 

First, the differences between the surveillance data and the data reported in the literature gathered by Matthews et al. (*19*) (and in our update in the online Technical Appendix) in terms of foodborne proportion suggests the possibility of publication bias in favor of foodborne outbreaks. This bias has been described; reports of larger foodborne outbreaks with severe outcomes are more likely to be published (*26*). This hypothesis is supported by the higher proportion of foodborne outbreaks caused by GII.4 and nonGII.4 found in the literature review.

Second, combining retrospective data from countries that differ in surveillance setup, coverage, and continuity is difficult. Individual cases of norovirus infection are not nationally notifiable in most of Europe, the United States, or New Zealand. Ascertainment and reporting of foodborne outbreaks probably varies substantially by setting (*27*) or between countries because of differing priorities and the complexity of the food chain. Despite this limitation, there was no feasible way to verify the transmission designation for every sample. Nevertheless, previous investigation of assignment of transmission modes within the FBVE network (*28*) showed that 2 of 13 countries used personborne transmission as a diagnosis of exclusion. Therefore, we consider that our estimate of the foodborne proportion is probably diluted and thus conservative. Ideally, data would be collected prospectively worldwide in a systematic approach, but until that is feasible, we consider our approach of using the data available from surveillance systems acceptable as best practice.

Third, differences in results based on P and C typing may reflect biases. This limitation mainly involves the FBVE/Noronet database, which includes data from multiple countries. For some countries, like the Netherlands, polymerase genotyping is the standard but capsid genotyping is performed for more thoroughly investigated outbreaks. Examples of reasons for additional capsid sequence typing are to find transmission chains (*29*,*30*), to confirm whether a new GII.4 variant is emerging (*22*), or to look for recombinants when suspected (*31*). Therefore, the FBVE/Noronet profiles based on capsid genotyping may be biased toward more unusual outbreaks and thereby possibly more often toward foodborne outbreaks. The FBVE network previously investigated the country-specific approaches (*27*,*28*) showing that, for example, Denmark has a surveillance system strongly focused on foodborne outbreaks, whereas in the United Kingdom, outbreaks in institutional settings are more often reported. This difference was reflected in the genotype profiles of these countries, thus confirming the different genotype profiles. In the United States, capsid sequencing is the standard, regardless of the outbreak circumstances; polymerase genotypes are not routinely uploaded to CaliciNet. In New Zealand, genotyping is based on the polymerase and capsid regions (if both can be determined), regardless of the outbreak circumstances. This systematic approach to genotyping regardless of the outbreak circumstances may explain why the US capsid profile and both the ESR-EpiSurv capsid and polymerase profiles are most similar to the FBVE/Noronet polymerase profile; these profiles are the result of systematic surveillance. A recent illustration of the value of performing both polymerase and capsid genotyping is the global emergence of the GII.4 Sydney 2012 variant (*32*). This variant is a GII.4 recombinant (*33*) with a GII.4 C-type and a GII.e P-type (*34*). Distinction of GII.4 versus nonGII.4 would not be possible if only polymerase-based genotype profiles were used.

Despite the shortcomings in working with surveillance data or data available in the public domain, our study showed that the proportion of norovirus outbreaks attributed to foodborne transmission is comparable in different parts of the world. The proportion of norovirus outbreaks attributed to foodborne transmission is in the same order of magnitude as the 17% found in an expert elicitation study from the Netherlands (*35*) and 11% in the United Kingdom as estimated from outbreak surveillance data (*36*). This similarity strongly indicates that this microbiology-based attribution method is robust, albeit in need of continued refinement (*6*). With �%^1 in 7 norovirus outbreaks being attributable to food, the foodborne transmission route represents a major target for intervention, particularly given the possibility of widespread exposures and the possibility of preventing not only primary but also secondary cases if contaminated foods are recalled from the market. Given the high incidence (*37*) and prevalence (*38*) of norovirus infections, norovirus has become a major cause of foodborne illness worldwide. To improve estimates of the social and economic costs of norovirus illness, future research should be aimed at filling the data gaps and should include nonindustrialized countries while aiming for global coverage of norovirus surveillance data.

Technical Appendix Proportion of foodborne outbreaks per norovirus genotype and results of systematic literature review.
