# Supplementary material for: Norovirus Genotype Profiles Associated with Foodborne Transmission, 1999�?"2012
Source: Emerg Infect Dis. 2015 Apr;21(4):592–9. doi: 10.3201/eid2104.141073 (PMC4378480; doi:10.3201/eid2104.141073)
Supplement: Technical Appendix — Proportion of foodborne outbreaks per norovirus genotype and results of systematic literature review. [file 14-1073-Techapp-s1.pdf]

Article DOI: http://dx.doi.org/10.3201/eid2104.141073

Norovirus Genotype Profiles Associated with Foodborne Transmission, 1999–2012

Technical Appendix

Technical Appendix Table. Proportion of foodborne outbreaks per genotype as estimated based on norovirus surveillance data in Noronet, ESR-EpiSurv, CaliciNet and an updated systematic review of scientific literature

Profile	FBVE/Noronet 1999-2012				ESR-EpiSurv 2008-2012				CaliciNet 2009-2012		Systematic literature review			
	P-type (n=4580)		C- type (n=2196)		P-type (n=685)		C-type (n=813)		C-type (n=3094)		P-type (n=107)		C-type (n=127)	
Group Type	N	Proportion FB (95%CI)	N	Proportion FB (95%CI)	N	Proportion FB (95%CI)	N	Proportion FB (95%CI)	N	Proportion FB (95%CI)	N	Proportion FB (95%CI)	N	Proportion FB (95%CI)
GI	315	0.36 (0.29-0.42)	169	0.53 (0.43-0.62)	56	0.28 (0.17-0.40)	66	0.28 (0.18-0.39)	322	0.30 (0.24-0.36)	15	0.71 (0.46-0.91)	14	0.91 (0.69-1.00)
I.1	12	0.17 (0.01-0.52)	10	0.57 (0.23-0.88)	0		0		4	0.25 (0.01-0.71)	0		0	
I.2	20	0.57 (0.32-0.81)	12	0.75 (0.43-0.96)	4	0.20 (0.01-0.61)	6	0.29 (0.05-0.65)	10	0.44 (0.15-0.75)	0		1	0.66 (0.15-0.99)
I.3	99	0.27 (0.18-0.38)	50	0.41 (0.26-0.58)	13	0.29 (0.09-0.54)	15	0.31 (0.12-0.55)	77	0.29 (0.18-0.41)	8	0.63 (0.29-0.90)	2	0.67 (0.15-0.99)
I.4	85	0.37 (0.25-0.50)	52	0.50 (0.35-0.66)	20	0.38 (0.19-0.59)	21	0.36 (0.18-0.57)	38	0.26 (0.11-0.45)	3	0.75 (0.28-0.99)	2	0.67 (0.16-0.99)
I.5	4	0.50 (0.10-0.90)	5	0.80 (0.39-0.99)	1	0.33 (0.01-0.84)	1	0.33 (0.01-0.85)	17	0.22 (0.07-0.43)	1	0.66 (0.15-0.99)	0	
I.6	0		32	0.56 (0.37-0.73)	14	0.27 (0.08-0.51)	21	0.23 (0.08-0.42)	150	0.29 (0.21-0.38)	1	0.33(0.01-0.84)	0	
I.7	12	0.50 (0.18-0.81)	7	0.57 (0.22-0.88)	0		0		24	0.60 (0.35-0.82)	0		0	
I.8	2	0.33 (0.01-0.84)	0		2	0.50 (0.09-0.91)	2	0.50 (0.09-0.91)	1	0.67 (0.16-0.99)	0		2	0.67 (0.17-0.99)
I.9	0		0		0		0		1	0.33 (0.01-0.84)	0		0	
I.14	0		0		0		0		0		0		3	0.67 (0.15-0.99)
I.a	2	0.75 (0.30-0.99)	n.a.		0		n.a.		n.a.		0		n.a.	
I.b	72	0.38 (0.26-0.50)	n.a.		2	0.25 (0.01-0.70)	n.a.		n.a.		0		n.a.	
I.d	4	0.60 (0.19-0.93)	n.a.		0		n.a.		n.a.		0		n.a.	
I.e	2	0.50 (0.09-0.90)	n.a.		0		n.a.		n.a.		0		n.a.	
I.f	1	0.67 (0.05-1.00)	n.a.		0		n.a.		n.a.		0		n.a.	
**GIInon4**	**656**	**0.28 (0.24-0.32)**	**467**	**0.44 (0.39-0.49)**	**71**	**0.31 (0.21-0.43)**	**190**	**0.28 (0.22-0.35)**	**602**	**0.25 (0.21-0.29)**	**23**	**0.56 (0.33-0.77)**	**46**	**0.82 (0.64-0.94)**
II.1	17	0.57 (0.32-0.80)	50	0.52 (0.38-0.67)	0		3	0.40 (0.07-0.80)	208	0.18 (0.13-0.24)	2	0.26 (0.01-0.71)	3	0.33 (0.01-0.84)
II.2	72	0.25 (0.14-0.38)	39	0.56 (0.39-0.72)	7	0.33 (0.09-0.65)	22	0.37 (0.16-0.53)	47	0.18 (0.07-0.32)	7	0.50 (0.15-0.85)	5	0.80 (0.39-0.99)

Profile	FBVE/Noronet 1999-2012				ESR-EpiSurv 2008-2012				CaliciNet 2009-2012		Systematic literature review			
	P-type (n=4580)		C- type (n=2196)		P-type (n=685)		C-type (n=813)		C-type (n=3094)		P-type (n=107)		C-type (n=127)	
Group Type	N	Proportion FB (95%CI)	N	Proportion FB (95%CI)	N	Proportion FB (95%CI)	N	Proportion FB (95%CI)	N	Proportion FB (95%CI)	N	Proportion FB (95%CI)	N	Proportion FB (95%CI)
II.3	5	0.33 (0.05-0.72)	142	0.21 (0.13-0.30)	0		48	0.22 (0.11-0.35)	22	0.31 (0.13-0.54)	10	0.62 (0.29-0.90)	11	0.80 (0.39-0.99)
II.5	5	0.20 (0.01-0.60)	3	0.80 (0.40-0.99)	1	0.67 (0.16-0.99)	2	0.50 (0.09-0.91)	4	0.80 (0.40-0.99)	2	0.50 (0.10-0.91)	3	n.a.
II.6*	2	0.25 (0.01-0.71)	93	0.53 (0.41-0.65)	1	0.66 (0.15-0.99)	59	0.20 (0.10-0.31)	128	0.28 (0.20-0.37)	0		7	0.67 (0.29-0.95)
II.7	195	0.27 (0.19-0.35)	71	0.64 (0.48-0.78)	3	0.50 (0.09-0.91)	27	0.41 (0.23-0.60)	66	0.29 (0.17-0.42)	1	0.33 (0.01-0.95)	7	0.86 (0.55-1.00)
II.8	9	0.50 (0.21-0.78)	4	0.80 (0.40-0.99)	0		0		0		0		2	0.67 (0.15-0.99)
II.10	0		1	0.33 (0.01-0.84)	0		0		0		0		1	0.67 (0.16-0.99)
II.12	14	0.46 (0.18-0.74)	30	0.46 (0.26-0.66)	16	0.31 (0.12-0.56)	17	0.53 (0.30-0.75)	98	0.42 (0.30-0.55)	1	0.67 (0.17-0.99)	0	
II.13	0		14	0.54 (0.28-0.79)	0		7	0.22 (0.03-0.54)	19	0.07 (0.00-0.25)	0		0	
II.14	0		9	0.38 (0.10-0.71)	0		1	0.67 (0.16-0.99)	3	0.50 (0.10-0.91)	0		1	n.a.
II.15	5	0.25 (0.01-0.71)	0		0		0		0		0		1	0.67 (0.16-0.99)
II.16	3	0.75 (0.29-0.99)	2	0.33 (0.01-0.84)	8	0.30 (0.07-0.60)	0		3	0.50 (0.10-0.90)	0		0	
II.17	8	0.50 (0.21-0.79)	2	0.25 (0.01-0.71)	0		0		3	n.a.	0		0	
II.20	4	0.33 (0.29-0.84)	6	0.38 (0.10-0.71)	4	0.34 (0.05-0.71)	4	0.34 (0.05-0.71)	0		0		0	
II.21	0		0		0		0		1	0.16 (0.05-0.32)	0		0	
II.22	2	0.75 (0.29-0.99)	0		1	0.33 (0.01-0.84)	0		0		0		0	
II.b	213	0.24 (0.18-0.30)	n.a.		14	0.13 (0.02-0.34)	0	n.a.	0		0		n.a.	
II.c	0		n.a.		14	0.57 (0.32-0.81)	0	n.a.	0		0		n.a.	
II.g	100	0.30 (0.21-0.40)	n.a.		3	0.40 (0.07-0.81)	**0**	n.a.	0		0		n.a.	
II.m	1	0.67 (0.15-0.99)	n.a.		**0**		**0**	n.a.	0		0		n.a.	
nonGII.4	971	0.30 (0.27-0.34)	636	0.46 (0.41-0.51)	127	0.29 (0.22-0.38)	256	0.29 (0.22-0.38)	924	0.26 (0.23-0.30)	38	0.63 (0.46-0.80)	60	0.87 (0.74-0.96)
GII.4***	3595	0.08 (0.07-0.09)	1528	0.19 (0.16-0.21)	547	0.09 (0.07-0.12)	546	0.09 (0.07-0.12)	2139	0.12 (0.10-0.14)	59	0.31 (0.18-0.45)	45	0.75 (0.52-0.92)
Mixed outbreak	14	0.71 (0.46-0.91)	31	0.81 (0.64-0.93)	11	0.46 (0.21-0.72)	11	0.46 (0.22-0.72)	31	0.16 (0.05-0.33)	10	0.63 (0.29-0.90)	22	0.88 (0.68-0.98)

* Region B does not well distinguish between the polymerase of GII.6/7/8.
** Estimates in green fonts do not statistically differ, i.e. 95% confidence intervals overlap, with FBVE/Noronet polymerase proportions as a reference; estimates given in red fonts do statistically differ.
***II.e is seen with the new variant II.4-Sydney capsid and thereby it is unclear whether this polymerase-based genotype should be considered a II.4 or II-non-4 genotype. This II.e polymerase genotype was included in the II.4 group in the polymerase profile, since it statistically showed most correspondence to the II-4 group.

Systematic Literature Review Update

1. Historical perspective: norovirus gastroenteritis outbreaks in military forces. Medical Surveillance Monthly Report. 2011;18:7–8. PubMed http://www.afhsc.mil/documents/pubs/msmrs/2011/v18_n11.pdf#Page=7

2. Outbreak surveillance. New Zealand Public Health Surveillance Report. 2011;9:5. https://surv.esr.cri.nz/PDF_surveillance/NZPHSR/2011/NZPHSR2011June.pdf

3. Outbreak case reports: Norovirus outbreak linked to consumption of imported raw oysters. New Zealand Public Health Surveillance Report. 2011;9:7–8.

4. Ahmed SF, Klena JD, Mostafa M, Dogantemur J, Middleton T, Hanson J, et al. Viral gastroenteritis associated with genogroup II norovirus among U.S. military personnel in Turkey, 2009. PLoS ONE. 2012;7:e35791. PubMed http://dx.doi.org/10.1371/journal.pone.0035791

5. Alfano-Sobsey E, Sweat D, Hall A, Breedlove F, Rodriguez R, Greene S, et al. Norovirus outbreak associated with undercooked oysters and secondary household transmission. Epidemiol Infect. 2012;140:276–82. PubMed http://dx.doi.org/10.1017/S0950268811000665

6. Arvelo W, Sosa SM, Juliao P, Lopez MR, Estevez A, Lopez B, et al. Norovirus outbreak of probable waterborne transmission with high attack rate in a Guatemalan resort. J Clin Virol. 2012;55:8–11. PubMed http://dx.doi.org/10.1016/j.jcv.2012.02.018

7. Baker K, Morris J, McCarthy N, Saldana L, Lowther J, Collinson A, et al. An outbreak of norovirus infection linked to oyster consumption at a UK restaurant, February 2010. J Public Health (Oxf). 2011;33:205–11. PubMed http://dx.doi.org/10.1093/pubmed/fdq089

8. Breitenmoser A, Fretz R, Schmid J, Besl A, Etter R. Outbreak of acute gastroenteritis due to a washwater-contaminated water supply, Switzerland, 2008. J Water Health. 2011;9:569–76. PubMed http://dx.doi.org/10.2166/wh.2011.158

9. Brucker R, Bui T, Kwan-Gett T, Stewart L, Hall AJ, Kinzer MH. Norovirus infections associated with frozen raw oysters - Washington, 2011. MMWR Morb Mortal Wkly Rep 2012;61:110. PubMed http://www.cdc.gov/mmwr/PDF/wk/mm6106.pdf

10. Carpentier M, Ollivro J, Baron R, Le Galudec M, Fauchier C, Minoui-Tran A, et al. Investigation and control of a nosocomial norovirus outbreak in a long-term care facility. Infect Control Hosp Epidemiol. 2011;32:1052–5. PubMed http://dx.doi.org/10.1086/662017

11. Chapman AS, Witkop CT, Escobar JD, Schlorman CA, DeMarcus LS, Marmer LM, et al. Norovirus outbreak associated with person-to-person transmission, U.S. Air Force Academy, July 2011. Medical Surveillance Monthly Report. 2011;18:2–5. PubMed http://www.afhsc.mil/documents/pubs/msmrs/2011/v18_n11.pdf#Page=2

12. Chitambar S, Gopalkrishna V, Chhabra P, Patil P, Verma H, Lahon A, et al. Diversity in the enteric viruses detected in outbreaks of gastroenteritis from Mumbai, Western India. Int J Environ Res Public Health. 2012;9:895–915. PubMed http://dx.doi.org/10.3390/ijerph9030895

13. Collier DA, Bayles MK, Barret JP. Acute gastroenteritis outbreak at the Armed Forces Retirement Home, Washington, DC, January 2011. Medical Surveillance Monthly Report. 2011;18:11–4. PubMed http://www.afhsc.mil/documents/pubs/msmrs/2011/v18_n06.pdf#Page=11

14. Cooper T, Atta M, Mackay A, Roberts H, Clement A. A major outbreak of Norovirus in an acute NHS hospital in 2010: a practical management approach. Journal of Infection Prevention. 2011;12:111–8. http://dx.doi.org/10.1177/1757177411402528

15. de Laval F, Nivoix P, Pommier de Santi V, Caballe D, Garnotel E, Maslin J. Severe norovirus outbreak among soldiers in the field: foodborne followed by person-to-person transmission. Clin Infect Dis. 2011;53:399–400. PubMed http://dx.doi.org/10.1093/cid/cir380

16. Di Bartolo I, Monini M, Losio MN, Pavoni E, Lavazza A, Ruggeri FM. Molecular characterization of noroviruses and rotaviruses involved in a large outbreak of gastroenteritis in Northern Italy. Appl Environ Microbiol. 2011;77:5545–8. PubMed http://dx.doi.org/10.1128/AEM.00278-11

17. Doménech-Sánchez A, Juan C, Perez JL, Berrocal CI. Unmanageable norovirus outbreak in a single resort located in the Dominican Republic. Clin Microbiol Infect. 2011;17:952–4. PubMed http://dx.doi.org/10.1111/j.1469-0691.2010.03411.x

18. Donia D, Kota M, Leno L, Ylli A, Cenko F, Divizia M. First outbreak of norovirus in Albania. Lett Appl Microbiol. 2011;53:283–7. PubMed http://dx.doi.org/10.1111/j.1472-765X.2011.03104.x

19. Gonzaga VE, Ramos M, Maves RC, Freeman R, Montgomery JM. Concurrent outbreak of norovirus genotype I and enterotoxigenic Escherichia coli on a U.S. Navy ship following a visit to Lima, Peru. [Electronic Resource]. PLoS ONE. 2011;6:e20822. PubMed http://dx.doi.org/10.1371/journal.pone.0020822

20. Guzman-Herrador B, Heier B, Osborg E, Nguyen V, Vold L. Outbreak of norovirus infection in a hotel in Oslo, Norway, January 2011. Euro Surveill. 2011;16:5–9. PubMed http://www.eurosurveillance.org/images/dynamic/EE/V16N30/V16N30.pdf

21. Hoefnagel J, van de Weerdt DH, Schaefer O, Koene R. A norovirus outbreak triggered by copper intoxication on a coach trip from the Netherlands to Germany, April 2010. Euro Surveill. 2012;17:20120301. PubMed

22. Kasper MR, Lescano AG, Lucas C, Gilles D, Biese BJ, Stolovitz G, et al. Diarrhea outbreak during U.S. military training in El Salvador. [Electronic Resource]. PLoS ONE. 2012;7:e40404. PubMed http://dx.doi.org/10.1371/journal.pone.0040404

23. Kirk MD, Lalor K, Raupach J, Combs B, Stafford R, Hall GV, et al. Food-and waterborne disease outbreaks in Australian long-term care facilities, 2001-2008. Foodborne Pathog Dis. 2011;8:133–9. PubMed http://dx.doi.org/10.1089/fpd.2010.0648

24. OzFoodNet Working Group. OzFoodNet quarterly report, 1 October to 31 December 2010. Commun Dis Intell Q Rep. 2011;35:29–37. PubMed

25. Laine J, Huovinen E, Virtanen MJ, Snellman M, Lumio J, Ruutu P, et al. An extensive gastroenteritis outbreak after drinking-water contamination by sewage effluent, Finland. Epidemiol Infect. 2011;139:1105–13. PubMed http://dx.doi.org/10.1017/S0950268810002141

26. Lee LE, Cebelinski EA, Fuller C, Keene WE, Smith K, Vinje J, et al. Sapovirus outbreaks in long-term care facilities, Oregon and Minnesota, USA, 2002-2009. Emerg Infect Dis. 2012;18:873–6. PubMed http://dx.doi.org/10.3201/eid1805.111843

27. Mayet A, Andreo V, Bedubourg G, Victorion S, Plantec J, Soullie B, et al. Food-borne outbreak of norovirus infection in a French military parachuting unit, April 2011. Euro Surveill. 2011;16:2–4. PubMed http://www.eurosurveillance.org/ViewArticle.aspx?ArticleId=19930

28. Nenonen NP, Hannoun C, Larsson CU, Bergstrom T. Marked genomic diversity of norovirus genogroup I strains in a waterborne outbreak. Appl Environ Microbiol. 2012;78:1846–52. PubMed http://dx.doi.org/10.1128/AEM.07350-11

29. Nguyen LM, Middaugh JP. Suspected transmission of norovirus in eight long-term care facilities attributed to staff working at multiple institutions. Epidemiol Infect. 2012;140:1702–9. PubMed http://dx.doi.org/10.1017/S0950268811002573

30. Nicolay N, McDermott R, Kelly M, Gorby M, Prendergast T, Tuite G, et al. Potential role of asymptomatic kitchen food handlers during a food-borne outbreak of norovirus infection, Dublin, Ireland, March 2009. Euro Surveill. 2011;16:10–15. PubMed

31. Partridge DG, Evans CM, Raza M, Kudesia G, Parsons HK. Lessons from a large norovirus outbreak: impact of viral load, patient age and ward design on duration of symptoms and shedding and

likelihood of transmission. J Hosp Infect. 2012;81:25–30. PubMed http://dx.doi.org/10.1016/j.jhin.2012.02.002

32. Repp KK, Keene WE. A point-source norovirus outbreak caused by exposure to fomites. J Infect Dis. 2012;205:1639–41. PubMed http://dx.doi.org/10.1093/infdis/jis250

33. Riera-Montes M, Brus Sjolander K, Allestam G, Hallin E, Hedlund KO, Lofdahl M. Waterborne norovirus outbreak in a municipal drinking-water supply in Sweden. Epidemiol Infect. 2011;139:1928–35. PubMed http://dx.doi.org/10.1017/S0950268810003146

34. Sarvikivi E, Roivainen M, Maunula L, Niskanen T, Korhonen T, Lappalainen M, et al. Multiple norovirus outbreaks linked to imported frozen raspberries. Epidemiol Infect. 2012;140:260–7. PubMed http://dx.doi.org/10.1017/S0950268811000379

35. Smith AJ, McCarthy N, Saldana L, Ihekweazu C, McPhedran K, Adak GK, et al. A large foodborne outbreak of norovirus in diners at a restaurant in England between January and February 2009. Epidemiol Infect. 2012;140:1695–701. PubMed http://dx.doi.org/10.1017/S0950268811002305

36. Sukhrie FHA, Teunis P, Vennema H, Copra C, Beersma MFCT, Bogerman J, et al. Nosocomial transmission of norovirus is mainly caused by symptomatic cases. Clin Infect Dis. 2012;54:931–7. PubMed http://dx.doi.org/10.1093/cid/cir971

37. Takanashi S, Wang Q, Chen N, Shen Q, Jung K, Zhang Z, et al. Characterization of emerging GII.g/GII.12 noroviruses from a gastroenteritis outbreak in the United States in 2010. J Clin Microbiol. 2011;49:3234–44. PubMed http://dx.doi.org/10.1128/JCM.00305-11

38. Thornley CN, Emslie NA, Sprott TW, Greening GE, Rapana JP. Recurring norovirus transmission on an airplane. Clin Infect Dis. 2011;53:515–20. PubMed http://dx.doi.org/10.1093/cid/cir465

39. Učakar V, Grilc E, Jeraj I. An investigation of a waterborne outbreak caused by microbiological contamination of the drinking water supply system. Slovenian Journal of Public Health. 2012;51:112–9. http://dx.doi.org/10.2478/v10152-012-0013-0 published online April 3.

40. Vantarakis A, Mellou K, Spala G, Kokkinos P, Alamanos Y. A gastroenteritis outbreak caused by noroviruses in Greece. Int J Environ Res Public Health. 2011;8:3468–78. PubMed http://dx.doi.org/10.3390/ijerph8083468

41. Vinnard C, Lee I, Linkin D. Successful control of a norovirus outbreak among attendees of a hospital teaching conference. Am J Infect Control. 2012;40:73–4. PubMed http://dx.doi.org/10.1016/j.ajic.2011.03.033

42. Wall R, Dymond N, Bell A, Thornley C, Buik H, Cumming D, et al. Two New Zealand outbreaks of norovirus gastroenteritis linked to commercially farmed oysters. N Z Med J. 2011;124:63–71. PubMed

43. Yang Z, Wu X, Li T, Li M, Zhong Y, Liu Y, et al. Epidemiological survey and analysis on an outbreak of gastroenteritis due to water contamination. Biomed Environ Sci. 2011;24:275–83. PubMed

44. Yap J, Qadir A, Liu I, Loh J, Tan BH, Lee VJ. Outbreak of acute norovirus gastroenteritis in a military facility in Singapore: A public health perspective. Singapore Med J. 2012;53:249–54. PubMed
